# A Cluster-Randomized Controlled Trial to Reduce Diarrheal Disease and Dengue Entomological Risk Factors in Rural Primary Schools in Colombia

**DOI:** 10.1371/journal.pntd.0005106

**Published:** 2016-11-07

**Authors:** Hans J. Overgaard, Neal Alexander, Maria Ines Matiz, Juan Felipe Jaramillo, Victor Alberto Olano, Sandra Vargas, Diana Sarmiento, Audrey Lenhart, Thor Axel Stenström

**Affiliations:** 1 Department of Mathematical Sciences and Technology, Norwegian University of Life Sciences, Norway; 2 Institut de Recherche pour le Développement, Maladies Infectieuses et Vecteurs, Ecologie, Génétique, Evolution et Contrôle, Montpellier, France; 3 Department of Entomology, Faculty of Agriculture, Kasetsart University, Bangkok, Thailand; 4 MRC Tropical Epidemiology Group, London School of Hygiene and Tropical Medicine, London; 5 Instituto de Salud y Ambiente, Universidad El Bosque, Bogota, Colombia; 6 Liverpool School of Tropical Medicine, Liverpool, United Kingdom; 7 U.S. Centers for Disease Control and Prevention, Atlanta, United States of America; 8 SARChI Chair, Institute for Water and Waste Water Technology, Durban University of Technology, Durban, South Africa; George Washington University School of Medicine and Health Sciences, UNITED STATES

## Abstract

**Background:**

As many neglected tropical diseases are co-endemic and have common risk factors, integrated control can efficiently reduce disease burden and relieve resource-strained public health budgets. Diarrheal diseases and dengue fever are major global health problems sharing common risk factors in water storage containers. Where provision of clean water is inadequate, water storage is crucial. Fecal contamination of stored water is a common source of diarrheal illness, but stored water also provides breeding sites for dengue vector mosquitoes. Integrating improved water management and educational strategies for both diseases in the school environment can potentially improve the health situation for students and the larger community. The objective of this trial was to investigate whether interventions targeting diarrhea and dengue risk factors would significantly reduce absence due to diarrheal disease and dengue entomological risk factors in schools.

**Methodology/Principal Findings:**

A factorial cluster randomized controlled trial was carried out in 34 rural primary schools (1,301 pupils) in La Mesa and Anapoima municipalities, Cundinamarca, Colombia. Schools were randomized to one of four study arms: diarrhea interventions (DIA), dengue interventions (DEN), combined diarrhea and dengue interventions (DIADEN), and control (CON). Interventions had no apparent effect on pupil school absence due to diarrheal disease (p = 0.45) or on adult female *Aedes aegypti* density (p = 0.32) (primary outcomes). However, the dengue interventions reduced the Breteau Index on average by 78% (p = 0.029), with Breteau indices of 10.8 and 6.2 in the DEN and DIADEN arms, respectively compared to 37.5 and 46.9 in the DIA and CON arms, respectively. The diarrhea interventions improved water quality as assessed by the amount of *Escherichia coli* colony forming units (CFU); the ratio of Williams mean *E*. *coli* CFU being 0.22, or 78% reduction (p = 0.008).

**Conclusions/Significance:**

Integrated control of dengue and diarrhea has never been conducted before. This trial presents an example for application of control strategies that may affect both diseases and the first study to apply such an approach in school settings. The interventions were well received and highly appreciated by students and teachers. An apparent absence of effect in primary outcome indicators could be the result of pupils being exposed to risk factors outside the school area and mosquitoes flying in from nearby uncontrolled breeding sites. Integrated interventions targeting these diseases in a school context remain promising because of the reduced mosquito breeding and improved water quality, as well as educational benefits. However, to improve outcomes in future integrated approaches, simultaneous interventions in communities, in addition to schools, should be considered; using appropriate combinations of site-specific, effective, acceptable, and affordable interventions.

**Trial Registration:**

ClinicalTrials.gov no. ISRCTN40195031

## Author Summary

Many tropical diseases co-occur in the same areas and have overlapping risk factors and strategies for control and prevention. Therefore, integrating several diseases in control activities can be both effective and cost-efficient. We evaluated sets of diarrhea and dengue interventions in rural primary schools in Colombia to reduce absence due to diarrheal disease among pupils and dengue risk factors. Integrating the control of these two diseases is justified because of the potential joint risk factor of water storage behavior. Stored drinking water may be contaminated with enteric pathogens and such containers also provide breeding opportunities for dengue vectors. Water storage is common in schools in many tropical countries. Although the study did not demonstrate clear evidence of a reduction in pupil absence due to diarrhea or reductions of adult mosquito densities in schools, significant reductions were detected in the number of mosquito breeding sites and drinking water quality was significantly improved. Integrated disease control interventions in school settings should be further explored to prevent diseases with overlapping etiologies amongst pupils and school staff.

## Introduction

Integrating the control of diseases can reduce their burden while relieving resource-strained public health budgets [1–3]. Many tropical diseases are co-endemic and have overlapping risk factors and strategies for control and prevention. Recent assessments of co-occurrence and co-infection of diseases have shown the potential for the integration of control and prevention strategies [4–8].

Diarrheal diseases and dengue fever co-occur in many parts of the world and water management practices can influence the number of infections of both. Storing water is a necessity in many places due to a lack of regular safe water supply. The storage of drinking water can be a determinant of both diseases if the stored water is fecally contaminated [9] and the containers used for storage provide breeding sites for dengue vector mosquitoes [10, 11]. Close to 90% of the global diarrheal disease burden is thought to be caused by unsafe water supply and lack of sanitation and hygiene [12, 13]. About 748 million people, 9% of the global population, lack access to safe water sources, of which >90% live in rural areas [14]. Dengue fever, caused by a flavivirus with four different serotypes, is the most common arboviral disease in the world [11]. Dengue is mainly transmitted by *Aedes aegypti*, which can also transmit chikungunya, Zika and yellow fever viruses [15]. Unplanned and unregulated urban development, poor water storage, and unsatisfactory sanitary conditions are all determinants of dengue transmission [17–21]. There are no theraputic drugs for dengue and although a recent licensed vaccine, Dengvaxia by Sanofi Pasteur, has been recommended by the World Health Organization (WHO) and is being rolled out in several countries [16], vector control will remain an important part of integrated control of dengue. Effective control of both of these diseases largely depends on the provision of a reliable supply of safe water, appropriate water management practices, and community participation in control efforts [13, 17, 18]. This functional relationship lends itself to integrated control approaches, which may be both efficient and cost-effective.

Although there are currently no analyses on the co-occurrence and co-infection of dengue and diarrheal pathogens, it is clear that both diseases individually are of great public health importance globally. More than 1.4 million deaths from diarrheal diseases were recorded in 2010, of which approximately 800,000 were children younger than 5 years [19, 20]. The annual number of deaths from dengue has been estimated at 14,000–22,000 [19, 21], mainly among children [21]. Approximately 2.5 billion people live in risk areas for dengue and an estimated 390 million infections occur annually in approximately 100 countries [17, 22, 23]. In 2010, more than 89 million disability-adjusted life years (DALYs) were estimated to be attributed to diarrheal disease and 825,000 DALYs attributed to dengue globally [24].

Both diarrhea and dengue are endemic throughout Latin America. Diarrhea is a leading cause of morbidity in Colombia and one of the ten most important causes of mortality [25]. The prevalence of diarrhea in children under five years old in 2010 was 13% [26]. Colombia has one of the highest levels of dengue transmission in the Americas. In 2014, dengue incidence was 413.5 cases per 100,000 inhabitants (110,473 cases and 294 deaths) [27]. About 50% of the urban population in the country is considered to be at high risk [28]. In the 2010 dengue epidemic, 85% of the cases came from municipal capitals, 8% from other population centers, and 7% from rural areas [29]. However, generally about 13–29% of dengue cases are reported from rural areas [30–32]. All four dengue virus serotypes circulate in the country and both *Ae*. *aegypti* and *Ae*. *albopictus* are present [33, 34]. *Aedes aegypti* is not only an urban species, but is also abundant in rural areas where it can have relatively high infection rates [35–37]. Inadequate safe drinking water supply and waste disposal services have been identified as principal drivers of *Ae*. *aegypti* propagation in Latin America [38, 39]. Storing water is common in Colombian households, even in areas with access to piped water. Water storage tanks, laundry basins (albercas), and drums are the primary dengue vector breeding sites in much of Colombia [40–42]. In 2009–2010, 63.5% of the population lacked access to water suitable for human consumption [43]. In 2012 the coverage of piped water was 97% in urban areas but only 53% in rural areas [44]. Lack of access to reliable, clean drinking water is likely a key factor in making diarrhea a leading cause of morbidity, particularly among children.

Disease risk for both dengue and diarrheal illnesses is often estimated by household-level variables, e.g. presence of mosquito positive containers and adult mosquitoes for dengue [11]; and lack of access to safe drinking water, inadequate sanitation and hygiene for diarrhea [13]. Control interventions for dengue and diarrhea often target households as well; e.g. insecticide treatment of containers, household repellents, window screening for dengue, and boiling or filtering of drinking water and improving toilets for diarrhea [11, 13]. However, children spend large portions of their days in school and could potentially be at risk of contracting illnesses while in the school environment. Only 54% of rural public schools in Colombia have access to drinking water, 57% to sewerage and 40% to a sufficient number of toilets [45]. For example, in the neighboring municipality of Apulo clean drinking water (absence of *E*. *coli*) was only available in 5 out of 14 schools (36%) [46], potentially exposing pupils to diarrheal pathogens from water ingested at school. Similarly, school children may be disproportionately exposed to mosquito bites, because the peak biting times of dengue vectors occur during school hours [42]. Educational interventions targeting schools to promulgate public health messages and engage students in practical control efforts are receiving increasing attention [47–49]. School children are responsive to public health education and may act as messengers of behavioral change to their households and communities [48, 50, 51].

The aim of this cluster-randomized controlled trial was to determine whether integrated interventions targeting determinants of diarrhea and dengue delivered to rural primary schools would reduce diarrheal disease, dengue entomological risk factors, school absenteeism due to illness, and contamination of stored water. A cluster design was used because the interventions are delivered at schools, with students nested within schools. Disease incidence and absence rates apply to both cluster and individual level, whereas entomological outcomes and water quality indicators pertain to the cluster level.

## Methods

### Setting and Participants

The study was undertaken in the municipalities of Anapoima and La Mesa in Cundinamarca department, Colombia. In 2011 Anapoima had a population of 12,539 inhabitants (57% in rural areas), a total area of 124.2 km^2^, an average altitude of 700 meters above sea level (m.a.s.l.), and an average temperature of 26°C [52]. The population for La Mesa in 2011 was 29,566 inhabitants (45% in rural areas), with a total area of 148 km^2^, an average altitude of 1,200 m.a.s.l. and an average annual temperature of 22°C [53]. The average annual rainfall in the area is 1,300 mm. The rainfall pattern is bimodal with a rainy peak in April-May, a relatively dry period in June-September and a second rainy peak in October-November. People cultivate crops; such as sugar cane, coffee, fruit; raise livestock, as well as work in tourism. Natural vegetation consists of dry tropical forest, premontane and lower montane moist forests.

**Fig 1:**
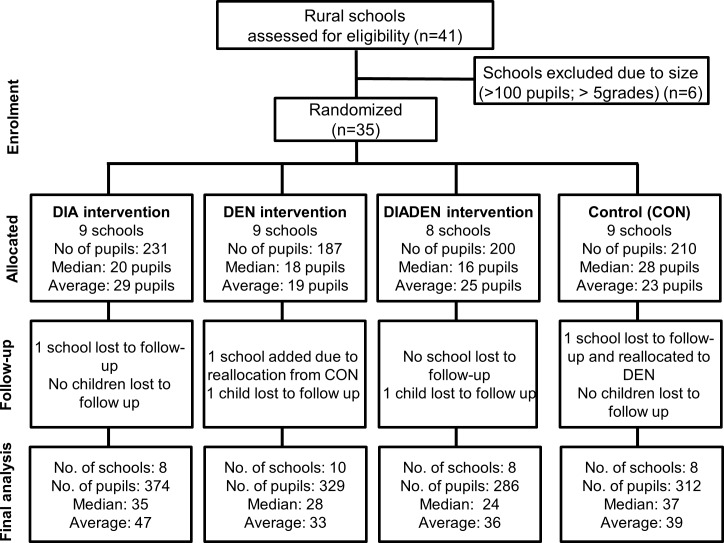
Flow diagram. Intervention assignment and follow-up analysis of schools and pupils in La Mesa and Anapoima municipalities in Cundinamarca department, Colombia.

We performed a 2×2 factorial cluster-randomized controlled trial in rural primary schools in the two municipalities. All rural primary schools were assessed for eligibility to participate in the trial. Inclusion and exclusion criteria for schools and pupils are described in detail in Overgaard *et al*. [54]. In brief, large schools (>100 pupils and >five grades) were excluded due to different teaching strategies, teacher-student dynamics, and number of pupils compared to primary schools. Thirty-five rural primary schools were randomized to the arms of the study (Fig 1). All pupils were eligible for inclusion. Those moving to a school outside the study area during the study were considered lost to follow up. However, those who moved to a school within the study area became part of the arm into which they moved.

### Interventions

Each school (cluster) was randomized to one of four study arms: 1) DIA, 2) DEN, 3) DIADEN, and 4) untreated control (CON). Interventions relating to either diarrheal diseases (DIA), dengue (DEN), or both (DIADEN) were implemented in the schools during February-April 2012. Interventions were implemented and generally maintained by the project team for two years (four complete school semesters). All materials were supplied by the project.

The DIA interventions–targeting drinking water quality, sanitation, and hygiene–consisted of installing water filters, fitting lids or nets on all drinking water storage containers to prevent contamination, and cleaning of the containers once per year. Lids and nets were inspected once or twice per month and improved if needed. Container cleaning was done once per semester by municipal workers. Hygiene practices included the promotion of hand washing with soap (before eating and after toilet visits) and proper use and daily cleaning of toilets. The DEN interventions–targeting adult and immature mosquito control and solid waste management–consisted of installing deltamethrin-treated curtains made from LifeNet material (Bayer CropScience) in classrooms and computer rooms, and fitting lids or nets on all water storage containers to prevent mosquito entry. Containers that could not be fitted with lids/nets, mostly *albercas* (laundry basins), were treated with pyriproxyfen (Sumilarv, Sumimoto Chemical Company), an insect growth regulator, which prevents the emergence of adult mosquitoes [55]. New pyriproxyfen was added to containers once every two months. Larval source management was carried out by pupils during weekly solid waste clean-up and collection campaigns. In addition, each set of interventions contained educational components consisting of project-designed educational teacher’s manuals and training guides adjusted to the curricula of children’s ages. The diarrhea educational component included lessons on symptoms, transmission pathways, risk factors, role of hand washing and hygiene, water and health relationships, etc. The dengue educational component included lessons on symptoms, transmission and risks, vector biology/ecology/control, the role of solid waste as mosquito breeding sites, etc. Every two months the project team met with teachers for training and delivery of new educational material. All DIA and DEN interventions pertained to the cluster level. Details of the interventions are provided in Overgaard *et al*. [54]. No dengue or diarrhea interventions by governmental or other actors were carried out in the rural schools during the study period.

Both the DIA and DEN interventions included lids or nets on containers. Since both target diarrhea and dengue outcomes, especially water quality and larval indices, they could have potential practical and statistical implications on the outcomes. This is discussed further in the Limitations section.

### Outcomes

The primary outcome measure for diarrheal disease (individual level outcome) was incidence rate of diarrhea in school children, assessed as the number of episodes (and days) students were absent due to diarrhea. The primary outcome measure for dengue entomological risk was the density of adult female *Ae*. *aegypti* mosquitoes per hour (Adult index), pertaining to the cluster level. The reason for selecting a mosquito index, and not a child health measure, as the primary dengue outcome is that, given the dengue incidence in the area, the required sample size would have been prohibitively large. Secondary outcome measures were the Breteau index (number of containers with *Ae*. *aegypti* immatures /100 schools), number of pupil absence episodes (and days) due to probable dengue and to any illness, and concentration of *Escherichia coli* in drinking water storage containers. Of the secondary outcomes, the Breteau index and level of *E*. *coli* contamination in water were cluster level outcomes, whereas pupil absence records were individual level outcomes but analyzed at the cluster level. Outcomes specific to the educational interventions will be presented in a separate publication.

### Sample size

Sample sizes were calculated incorporating both the diarrhea and dengue primary outcome indicators. The sample size calculation based on diarrhea data considered both the number of schools and number of children per school. For dengue, the sample size was determined in terms of numbers of schools, since this outcome was only measured at the school level. Calculations were carried out using a target number of participants of 873 pupils (data from 2006–2007) from 35 schools with an average of 25 pupils per cluster (school) (range: 5–96). The harmonic mean of 17 children per school was used in calculations to allow for school size variation. The sample size for the primary outcome of diarrhea incidence was calculated using methods for cluster-randomized trials [56]. The baseline diarrhea incidence (0.28/person-year) and within-school clustering (coefficient of variation *k* = 0.8) was calculated from existing data from the study area (Instituto de Salud y Ambiente, El Bosque University, Bogotá). For 17 children per school followed up for two years, 35 schools achieved 90% power for a 75% reduction in incidence and 5% two-sided significance level, or 80% power for a 65% reduction. For the sample size calculation for the dengue endpoint, we used data on *Ae*. *aegypti* adult density from Mexican schools [57], since no comparable data were available from Colombia. A negative binomial distribution was fitted to these data to allow for overdispersion relative to Poisson, giving a mean of 24 mosquitoes per school and a dispersion parameter of 0.75. Power was estimated assuming equal numbers of mosquitoes per arm [58]. Using these parameters and 17 schools per arm, a 70% reduction in mosquito numbers was detectable with 84% power and a 75% reduction was detectable with 92% power.

### Sequence generation and allocation concealment

Schools (clusters) were allocated to trial arms at a public randomization event in each municipality before the 2012 school year, which started in February. At each event, a raffle was arranged by project investigators at El Bosque University where a representative of each school drew a number indicating to which arm their school would be allocated. This method maintained allocation concealment, i.e. the investigators and participants were ignorant of the upcoming assignment of each school. However, the assignment was not blind. The achieved allocation ratio was 9:9:8:9 schools and 231:187:200:210 pupils in the DIA:DEN:DIADEN:CON arms, respectively (Fig 1). All pupils in each cluster were included in the study, i.e. complete enumeration of participants. Allocation was stratified by municipality [59], because the two municipalities differed in ways, which were likely to be associated with the trial outcomes. In particular, the La Mesa schools are located at higher altitudes (712–1610 m.a.s.l.) than the ones in Anapoima (588–1089 m.a.s.l.) and only Anapoima has a municipal educational board, potentially improving educational follow-up.

### Data collection

Baseline data were collected during July-September 2011 (dry season) and October-November 2011 (rainy season). All data collections and follow-up of interventions were done by the project teams at four time points after implementation of interventions, May-June 2012, October-November 2012, May-June 2013, and October-November 2013. Absences were recorded daily by teachers and every week absence records were collected by project staff. An absence episode was defined as the absence of a pupil for all or part of a school day. Absences for health reasons were confirmed by phone calls to parents and, if necessary, house visits. The condition of the child was verified by the project physician or project staff by confirming symptoms and disease criteria. Diarrhea was defined as the passage of three or more loose or liquid stools per day (or more frequent passage than is normal for the individual) [60]. A new absence episode due to diarrhea was defined as one occurring after at least three consecutive diarrhea-free days [61]. This 3-day criterion was also used for any absence reason. Probable dengue was defined according to WHO criteria [11].

Adult mosquito collections were carried out inside schools using a battery-driven Prokopack aspirator [62] for 10 minutes in each classroom and computer room. Immature mosquito collections were carried out in all artificial and natural water holding containers within the perimeter of each school property (maximum ~80 m). Mosquitoes were identified to species in a field laboratory using common identification keys [63, 64].

A 200 mL water sample was collected from each drinking water container (storage tanks, water stored in the filter, tap water after filtration, and unfiltered water from taps). *Escherichia coli* presence was used as an indicator of fecal contamination and risk of diarrheal illness according to WHO guidelines [9]. Between 24 to 48 hours after collection, water samples were analyzed for *E*. *coli* using the analytical method 9222 B described in Eaton *et al*. [65]. Results were read between 24–48 hours and recorded as colony forming units (CFU)/100mL. All water analyses were carried out at Daphnia Laboratory, Bogotá, Colombia (certified laboratory by IDEAM, Ministry of Environment and Sustainable Development, Res. 0347/2010 and 0710/2012).

### Statistical methods

The primary diarrhea outcome, diarrhea incidence in school children, was expressed, for each school, as the incidence of episodes of school absence ascribed to diarrhea per school year. Incidence was also calculated in terms of numbers of absence days. These rates, and those of other causes of absence, were calculated per year based on a school year of 185 days. Analysis of covariance (ANCOVA) was used to estimate the effect of the diarrhea interventions on these absence rates. The factorial design was represented by including one dichotomous explanatory variable for each of the two interventions. The stratification was represented by including a further such variable for municipality.

The primary entomological outcome, adult female *Ae*. *aegypti* density (Adult Index), was expressed as mean number of adult female mosquitoes collected per hour averaged over the four collection times. A negative binomial regression model was used to analyze number of mosquitoes with the logarithm of the sampling effort (i.e. person-time spent aspirating) as an ‘offset’. This analysis yielded density ratios. As for the diarrhea outcome, the explanatory variables for the primary analysis were trial arm and stratum. Secondary analyses were carried out for both outcomes including another binary explanatory variable representing the interaction term between the two interventions.

The Breteau index (BI: number of containers with *Ae*. *aegypti* immatures/100 schools), was calculated at baseline as well as for each follow up time point. Four additional entomological variables were calculated: the School Index (SI: number of schools with *Ae*. *aegypti* immatures/schools inspected × 100), the Container index (CI: number of containers with *Ae*. *aegypti* immatures/containers inspected × 100), pupae per person (number of *Ae*. *aegypti* pupae/person), and the proportion of schools with adult female *Ae*. *aegypti* (%). These indices were analyzed similarly to the Adult Index. For BI and SI, the denominator for each school was the number of times it was sampled in the intervention period. For CI it was the total number of containers inspected, and for pupae per person it was the number of persons present per school—children plus teachers and other staff—at the end of 2012, multiplied by the numbers of times the school was sampled.

Although not pre-specified, the above analyses of the Adult and Breteau indices were repeated including the respective baseline values as an additional covariate. Due to the skewness of the values, this was done by categorizing the index as zero, or above or below the median of the positive values.

The percentage of *E*. *coli* positive water samples taken from water containers and mean *E*. *coli* concentration per sample (based on all samples, including negatives, expressed as colony forming units, CFU/100mL) were compared between arms using factorial analysis of covariance as for diarrhea incidence. Due to the skewness of CFU counts, the Williams mean (WM) CFU was calculated, i.e. 1 was added to all counts and the geometric mean was calculated, then 1 was subtracted again. This was done for all sampled containers over all surveys in the intervention period. The logarithm of WM for each school was used as the response variable in the analysis, and the coefficients anti-logged to give results in terms of ratios of WM.

The effect of duplicating interventions (lids / nets on containers) in both the DIA and the DEN interventions was not explicitly analyzed. Instead, the interpretation of the outcomes should be as follows: When comparing DIA against DEN, the shared interventions (lids / nets) are effectively not evaluated. When comparing DIADEN and DEN, only the additional DIA interventions are effectively compared with the non-DIA interventions, and the shared ones are not evaluated. A similar argument as the previous one applies for the DIADEN and DIA comparison.

### Consent and ethical considerations

The scope and objectives of the project were presented to the mayors and the secretaries of education and health in the two municipalities. The project was then presented to school principals and teachers who signed consent to participate (before randomization) on behalf of each school. The study was approved by the Comité Institucional de Ética en Investigaciones de la Universidad El Bosque, Bogotá, Colombia (Acta No. 146 of 30/08/2011) and the Ethical Review Board of London School of Hygiene and Tropical Medicine (Ref. no. 6289). The trial protocol was reviewed by the Regional Committees for Medical and Health Research Ethics (REC) in Norway. Pupils with written or oral assent and written or oral parental consent were to be included in the study. Written consent and assent were documented from the majority of parents and pupils. Parental consent was sought via information and consent forms which pupils were asked to take home. Some forms were mislaid and, on belatedly collating the returned ones, many were found to be illegible, or unidentifiable for other reasons such as the names being incomplete or at variance with those in our records. However, oral consent from parents or guardians were sought during telephone calls when establishing reasons for student’s school absence. Bearing in mind that the study was minimal risk in the terms of the Colombian Ministry of Health’s Resolution 8430 of 1993, we sought and received permission from the ad-hoc ethical committee of the Universidad El Bosque (Acta No. 009 of 27/11/2014) and the Ethics Committee of the London School of Hygiene and Tropical Medicine (reference 10453/6289, 7 March 2016) to publish all data collected. Both ethics committees approved the described consent procedures.

The trial is registered in the Current Controlled Trials (no. ISRCTN40195031).

## Results

### Participant flow

In December 2011, 35 schools were randomized to four study arms with nine schools in each of the DIA, DEN, and CON arms; and eight in the DIADEN arm (Fig 1). At the start of the trial, there were 828 pupils in these schools, with a total of 941 pupils participating in 2012 and 948 pupils in 2013. The total number of pupil observation days was 287,578. One school in the DIA arm in La Mesa was closed in the end of 2011 due to unstable ground conditions and was treated as lost to follow up. Another school in the DEN arm in Anapoima was closed in 2012 (after the first semester of interventions) due to structural damage to the building and the pupils in this school were moved to the closest available school. Since the closest school was in the CON arm and the transferred pupils had already started receiving the DEN interventions, this school was moved to the DEN arm. After reconstruction of the first school, the pupils returned there, resulting in both schools remaining in the DEN arm for the duration of the study.

### Baseline characteristics

There were only minor differences between schools in general baseline characteristics (Table 1). The altitudinal range of schools in the DEN and CON arms was high compared to the other arms. Entomological indices were generally higher during the rainy season. There were no major differences in entomological indices between arms, apart from slightly higher larval indices in the DIADEN schools. Schools in the DIADEN arm seemed to rely relatively more on rainwater rather than piped water and had a less frequent daily water supply compared to the other schools. A high proportion of schools across arms used boiling as a main water treatment method. Water contamination was quite similar across arms with 56–76% of samples containing *E*. *coli* and no significant differences between contamination levels (Table 1).

**Table 1:** Baseline characteristics of 35 rural primary schools allocated to four arms receiving either diarrhea (DIA), dengue (DEN), both (DIADEN), or no interventions (CON) in Anapoima and La Mesa municipalities, Colombia in 2011.

Variable	DIA	DEN	DIADEN	CON
**Number of schools**	**9**	**9**	**8**	**9**
**General**				
Altitudinal range (m.a.s.l.)	908–1350	712–1610	592–1093	588–1569
Total no. of teachers	13	10	10	11
Pupils per teacher	17	19	19	19
Total no. of classrooms (incl. computer rooms)	39	39	41	38
Male/female ratio	1.2	1.2	1.3	1.4
Mean pupil age (in years) (SD)	8.5 (2.2)	8.2 (2.2)	7.9 (2.1)	8.0 (2.2)
Age range	5–16	4–13	4–13	5–14
Degree of isolation (based on no. of nearby houses)^a^				
No. of completely isolated schools	1	1	1	3
No. of schools with a few (1–5) nearby houses	5	8	7	4
No. of schools with many (>5) nearby houses	2	1	0	1
**Entomological indices (dry / rainy season)**^**b**^				
School Index, SI (%)	12.5 / 12.5	20.0 / 10.0	25.0 / 37.5	12.5 / 25.0
Container Index, CI (%)	2.5 / 6.1	4.4 / 3.1	9.4 / 6.9	3.2 / 10.0
Breteau Index, BI	12.5 / 37.5	20.0 / 20.0	37.5 / 50.0	12.5 / 62.5
Pupae per person	0 / 0.004	0 / 0	0.03/ 0.005	0.03 / 0.09
Proportion of schools with female *Ae*. *aegypti* (%)	12.5 / 62.5	22.2 / 22.2	50.0 / 62.5	44.4 / 44.4
Adult index (female *Ae*. *aegypti*/hour)	1.05 / 0.71	0.53 / 0.47	0.73 / 2.79	0 / 1.12
**Water source**				
Schools with municipal water supply connection (%)	8	19	13	10
Schools with local water supply connection (%)	74	66	32	53
Schools using rain water (%)	33	25	71	40
Schools with daily water supply^c^ (%)	54	34	29	50
Schools that boil drinking water (%)	80	85	90	97
Schools with water connection in kitchen (for food preparation) (%)	92	75	84	90
Schools with water connection in sinks (for hand washing and drinking purposes) (%)	44	77	71	83
Schools with water connection in toilets (%)	77	63	52	83
**Water quality**^**d**^				
Samples *E*. *coli* positive (%)	56	59	76	76
Mean *E*. *coli* concentration (log10CFU+1)/100mL) (95% CI)	1.2 (0.6–1.7)	1.7 (1.0–2.4)	1.2 (0.7–1.6)	0.9 (0.5–1.3)

^a^ Degree of isolation. Number of nearby houses, i.e. within approximately 100 m from the perimeter of the school. One school in the DIA arm was closed in the beginning of the project and was removed. Therefore, the number of schools in the arm does not correspond to the original number.

^b^ Dry season: July-September 2011, rainy season: October-November 2011. School Index = % of schools positive of immature *Ae*. *aegypti*, Container Index = % of containers positive of immature *Ae*. *aegypti*, Breteau Index = no. of containers positive of immature *Ae*. *aegypti* per 100 schools, Pupae per person = no. of *Ae*. *aegypti* pupae per person.

^c^ A daily water supply could also mean supply during parts of the day.

^d^ Water samples collected in May-Jun 2011 from all drinking water tanks and taps (in kitchen, sinks, and water storage tanks).

SD = standard deviation. CFU = colony forming units. CI = confidence interval.

### Outcomes

#### General school absence and absence reasons

A total of 1,301 pupils were followed during the study period, 772 (59.3%) in La Mesa and 529 (40.7%) in Anapoima. Some of the 941 pupils in 2012 and 948 pupils in 2013 attended these schools during both years, but others only during one year. There were 7,850 general absence episodes, of which 4,836 (61.6%) were in La Mesa and 3,014 (38.4%) in Anapoima. This corresponds to 7,722 absence days in La Mesa (64%) and 4,329 absence days in Anapoima (36%). Overall, general school absence due to any reason varied from 4.4 to 5.5 episodes per pupil per year and 6.2 to 8.9 days per pupil per year across arms with no significant differences between arms. There were no significant differences between municipalities in mean number of absence episodes (Anapoima: 5.3 episodes/pupil/year; La Mesa: 5.4 episodes/pupil/year) and days (Anapoima: 7.5 days/pupil/year; La Mesa: 9.2 days/pupil/year). The most common single reason for absence was illness, accounting for about 21–28% of all absences (Table 2). Of those pupils that were absent due to illness, the most frequent symptoms were cold (38%), non-specific fever (10.3%), diarrhea (7.8%), and stomach pain and vomiting (7.1%). Other illness absences were injuries (6.2%), headaches (5.3%), indigestion (3.3%), enflamed throat (2.9%), dental problems (2.7%), ear infection/ear pain (2.2%), asthma (1.9%), dengue (1.5%), skin problems (1.4%), and chicken pox (1.2%).

**Table 2:** Pupil school absence (%) in rural primary schools in four arms receiving either diarrhea (DIA), dengue (DEN), both (DIADEN), or no intervention (CON) in Anapoima and La Mesa municipalities, Colombia, 2012–2013.

Absence reason	DIA	DEN	DIADEN	CON
**Total number of episodes**	**2,250**	**1,924**	**1,554**	**2,122**
Illness	24.5	21.4	28.4	24.6
Family reasons^a^	13.1	17.0	13.9	16.5
Travel (out of study area)	13.7	14.3	11.7	12.4
Medical / dental appointment	15.2	12.5	18.1	9.7
Lack of motivation	10.0	13.8	8.6	13.2
Transport problems^b^	11.2	10.1	8.0	10.3
No information on reason given	3.3	2.7	3.1	1.9
Inadequate school uniform / suspended	1.3	1.6	1.5	3.5
Other^c^	7.6	6.5	6.5	7.8

^a ^Family commitments, sick mother, no-one to accompany the child to school, taking care of home or siblings, bereavement or other family misfortune, family dysfunction.

^b^ Adverse weather conditions, distance to school, lack of money for transport.

^c^ Including: overslept, stayed at grandparents, move to another house, religious festivities, did not know there was class, adapting to school (for very small children).

### Primary outcomes

#### Diarrhea incidence rate (school absence rate due to diarrhea)

The overall incidence rate of absence episodes due to diarrhea was between 0.08 and 0.13 per pupil per year, across the arms. The effect of the diarrhea interventions on this incidence was small (0.03 episodes/pupil-year, Table 3) and not statistically significant (95% confidence interval: -0.05–0.12, p = 0.45). Analysis in terms of days rather than episodes led to similar conclusions (Table 3).

**Table 3:** Effect on primary and secondary outcomes of respective intervention in rural primary schools in arms receiving either diarrhea (DIA), dengue (DEN), both (DIADEN), or no intervention (CON) in Anapoima and La Mesa municipalities, Colombia, 2012–2013.

	DIA	DEN	DIADEN	CON	Effect of intervention of interest^a^(95% CI), p-value	
Number of schools	8	10	8	8	Diarrhea	Dengue
**Primary outcomes**						
**Mean (range) school absence in pupils per year due to diarrhea**						
**Episodes**^b^	0.13	0.12	0.13	0.08	0.03	
	(0–0.36)	(0–0.37)	(0.03–0.49)	(0–0.27)	(-0.05–0.12),	
					0.45	
**Days**	0.23	0.17	0.33	0.22	0.09	
	(0–0.60)	(0–0.52)	(0.03–0.98)	(0–0.79)	(-0.1–0.28),	
					0.32	
**Adult Index** (female *Ae*. *aegypti*/hour)^c^	1.37	1.38	2.27	1.79		1.01
	(0–6.41)	(0–6.78)	(0.22–6.44)	(0–7.40)		(0.23–4.39)
						0.99
**Secondary outcomes**						
**Mean (range) school absence in pupils per year due to dengue**						
**Episodes**	0.01	0.02	0.02	0.02		0.001
	(0–0.05)	(0–0.07)	(0–0.09)	(0–0.10)		(-0.02–0.02),

						0.93
**Days**	0.05	0.05	0.09	0.12		-0.01
	(0–0.25)	(0–0.37)	(0–0.52)	(0–0.79)		(-0.13–0.10),
						0.81
**Breteau Index** (no. positive containers per 100 schools)	37.5	10.8	6.25	46.9		0.22
	(0–150)	(0–50)	(0–25)	(0–150)		(0.06–0.84),
						0.03

^a^ Rate difference for absenteeism outcomes; rate ratio for entomological outcomes

^b^Between-cluster coefficient of variation = 1.0

^c^Between-cluster coefficient of variation = 1.3

CI = confidence interval.

### Density of adult female *Ae*. *aegypti* per school

The mean density of adult female *Ae*. *aegypti* mosquitoes varied from 1–2 per hour and there were no significant differences between arms (Table 3). Similarly, exploratory analysis showed no significant differences between arms comparing female *Ae*. *aegypti* density in classrooms (p = 0.89), toilets (p = 0.24), canteens (p = 0.68), kitchens (p = 0.51), or teacher’s bedrooms (p = 0.21).

### Secondary outcomes

#### Breteau Index and other entomological indices

**Fig 2:**
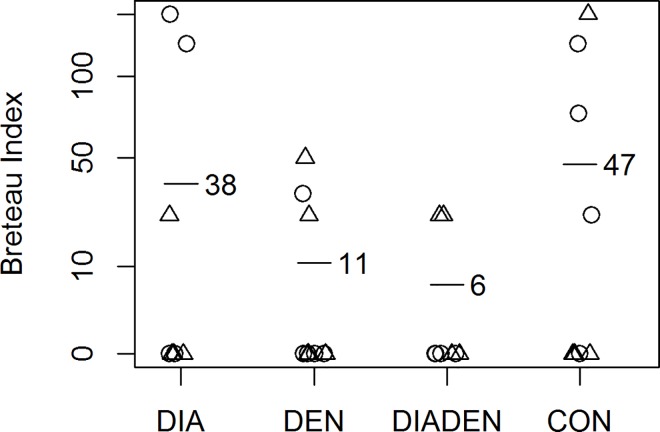
Effect of interventions on Breteau indices. Mean Breteau indices (horizontal bars with number) in rural primary schools in four arms receiving either diarrhea (DIA), dengue (DEN), both (DIADEN), or no intervention (CON). Each plot symbol is one school (circles = Anapoima, triangles = La Mesa. The dengue interventions reduced the mean Breteau Index by 78% (ratio 0.22, 95% confidence interval 0.06–0.84, p = 0.029).

**Fig 3:**
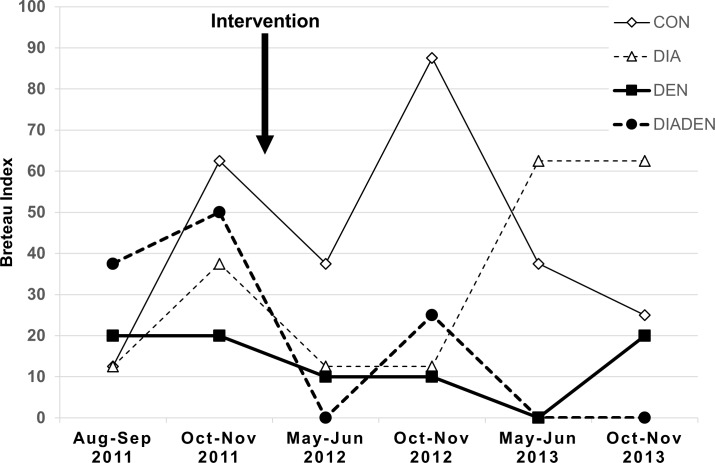
Breteau indices over time. Variation in Breteau index in rural primary schools in four arms receiving either diarrhea (DIA), dengue (DEN), both (DIADEN), or no intervention (CONTROL) in Anapoima and La Mesa municipalities, Colombia. Rainfall is bimodal with peaks in April-May and October-November.

The dengue interventions reduced the mean Breteau Index by almost 80% (ratio 0.22, 95% confidence interval 0.06–0.84, p = 0.029) (Fig 2). The lowest BI of 6.25 was in the DIADEN arm, followed by the DEN arm with a BI of 10.8 with the DIA and CON arms having significantly higher values (Table 3). There was no evidence that the effect of the dengue interventions differed according to the presence or absence of the diarrhea interventions (p value for interaction 0.87). The DIADEN arm had lower BI on average than the DEN arm but, in exploratory analysis, the ratio of 0.61 was not statistically significant (95% confidence interval 0.07–5.62, p = 0.65). The BI’s varied between years, but a potential effect of the interventions can be observed in the DEN and DIADEN arms following implementation of interventions (Fig 3). The other immature indices showed similar patterns (Table 4). In particular, there were fewer pupae per person in the dengue intervention arms: the ratio estimated by regression was 0.058, or a reduction of about 94%, but this was not statistically significant (95% confidence interval for the ratio 0.002–2.04, p = 0.11).

**Table 4:** Outcomes of entomological indices, values are mean (range) of values over four collection points, in rural primary schools in study arms receiving diarrhea (DIA), dengue (DEN), both (DIADEN), or no intervention (CON) in Anapoima and La Mesa municipalities, Colombia, 2012–2013.

Index	DIA	DEN	DIADEN	CON
Number of schools	8	10	8	8
School index, SI (%)	21.9 (12.5–37.5)	10.5 (0–20)	6.5 (0–25)	27.3(22.2–37.5)
Container index, CI (%)	7.5 (2.1–18.5)	2.2 (0–5.4)	1.1 (0–3.8)	7.1 (5.3–13.5)
Pupae per person^a^	0.18 (0.005–2.2)	0.04 (0–0.7)	0.05 (0–0.6)	0.36 (0.3–2.6)
Proportion of schools with female *Ae*. *aegypti* (%)	34.4 (25.0–50.0)	31.6 (20.0–44.4)	51.6 (37.5–75.0)	42.4 (25.0–50.0)

^a^Theoretical threshold levels of 0.5–1.5 *Ae*. *aegypti* pupae per person considered risk for dengue transmission [66].

Including the baseline BI in the analysis did not substantially change the results: the ratio for the dengue interventions was 0.26 rather than 0.22, and the p value 0.025 rather than 0.029. For Adult Index, one school could not be included due to lack of baseline data but, in the remainder, inclusion of the baseline values changed the estimated intervention effect from a null value (ratio of 1.01) to a detrimental one (2.7) and the p value decreased from 0.99 to 0.04.

### Other school absence

The overall total absence due to any causes of illness were 1,935 episodes and 3,569 days with a mean rate of 1.2 episodes/pupil/year (range: 1.0–1.2) and 2.3 (range: 1.9–2.7) days/pupil/year. These absence rates were similar between municipalities and there was no significant effect of either set of interventions.

There were 10 cases of probable dengue in 2012 and 19 in 2013. The mean between-arm rates of probable dengue varied between 0.01–0.23 episodes/pupil/year and 0.05–0.12 days/pupil/year. There were no significant differences between arms in absence episodes (p = 0.97) or number of absent days (p = 0.77) due to probable dengue. La Mesa had a significantly higher number of absence episodes and days due to probable dengue compared to Anapoima (La Mesa: 0.03 episodes, Anapoima: 0.019 episodes, p = 0.03; La Mesa: 0.15 days, Anapoima 0.008 days, p = 0.047).

### Water quality

**Fig 4:**
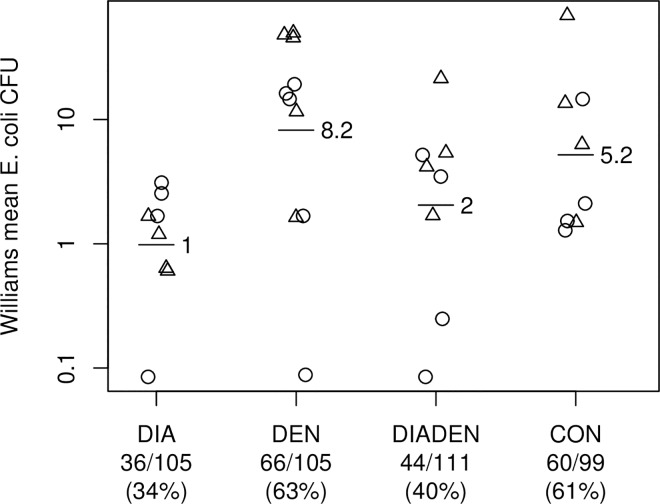
Effect of interventions on water quality. Mean *E*. *coli* concentration in drinking water storage containers, taps and water filters (horizontal bars with number) in rural primary schools in four arms receiving either diarrhea (DIA), dengue (DEN), both (DIADEN), or no intervention (CON). Each plot symbol is one school (circles = Anapoima, triangles = La Mesa). Numbers below graph show no. of *E*. *coli* positive containers/no. of sampled containers (percent positive containers). The diarrhea interventions reduced the Williams mean colony forming units (CFU) by 78% (ratio 0.22, 95% confidence interval 0.07–0.65, p = 0.008).

A total of 420 water samples were collected from water storage tanks (n = 159), taps (n = 138), water filters (n = 86), and boiled water (n = 37). The percentage of *E*. *coli* positive samples and mean *E*. *coli* concentration was significantly lower in the DIA and DIADEN arms (34% and 40%, respectively) than in the DEN and CON arms (63% and 61%, respectively) (p<0.05) (Fig 4). On average the diarrhea interventions reduced the Williams mean *E*. *coli* CFU by 78% (ratio 0.22, 95% confidence interval 0.07–0.65, p = 0.008) compared to schools which did not receive these interventions. Twelve of the 16 DIA and DIADEN schools (75%) were free of *E*. *coli* contaminated water in filtered water samples.

### Combination effect of duplicating single interventions

Although the study was designed to evaluate the overall effect of each of the two sets of disease-specific interventions and not specific single interventions within these sets, it is important to consider the duplication of container lids / nets on the outcomes. When interpreting these results (see Limitations section) one must recognize that in any DIA vs. DEN comparison the lids and covers will not count as these are shared between the two interventions.

### Harms

Some minor adverse reactions, possibly arising from contact to insecticide-treated curtains, were noted during the installation of curtains and first year of the study. Of the 400 pupils, 21 teachers, 11 project staff, 4 tailors, and a few others who were exposed to the nets 16 developed slight allergic reactions. Of these, 7 were project staff, 4 tailors, 2 pupils, 2 teachers, and a housewife. The most common symptoms were slight numbness, skin reactions, and itching, which usually resolved after 24–48 hours. None of the cases required medical examination. No further adverse reactions were noted during the remainder of the project.

## Discussion

This is the first trial in which interventions targeting dengue and diarrheal disease have been combined in an attempt to reduce disease incidence and risk factors. It is also the first trial to study the application of these combined interventions in school settings. Although there were no significant differences between study arms for the primary outcome indicators, school absence due to diarrhea disease and density of adult female *Ae*. *aegypti* mosquitoes, differences in secondary outcome indicators, including Breteau Index (BI) and water quality, suggest intervention impact.

### Dengue

The efficacy of the dengue interventions is clearly shown by the 78% reduction in immature *Ae*. *aegypti* infestation (BI) in schools that received dengue interventions, either alone (DEN) or in combination (DIADEN) compared to the DIA and CON arms. The DIADEN arm had a mean BI of 6.5 (positive containers per 100 schools) and the DEN arm 10.5. The lower BIs show that targeting larval breeding sites with covers, container-cleaning, pyriproxyfen, and residual waste clean-up campaigns resulted in reduced larval breeding in these school settings. Recent trials have reached similar reductions in BI’s. For example, BI was reduced by 57% through implementation of insecticide-treated curtains and water container covers in Colombia [67], by 65% through pesticide-free evidence-based community mobilization interventions in Mexico and Nicaragua [18], and by 86% through community-based control including container covers, health education, and garbage clean-up campaigns in India [68]. Overall BIs were exceptionally high in the study area, reaching values close to 90 (Fig 3). This is much higher than a BI of 5 which has been considered a threshold for disease transmission [69]. However, the BI and other *Stegomyia* indices (HI and CI) have been shown not to consistently reflect dengue transmission risk [70]. For example, dengue transmission occurred frequently in several studies even though the BI was lower than the proposed threshold value of 5 (summarized in [70]). Other indices such as adult or pupal indices have been proposed as better indicators of dengue transmission risk as they more accurately reflect the adult stage when mosquitoes can be infectious [71]. The observed differences in BI are also reflected in the other immature indices, but not in the adult indices (Table 4). In fact, adjusting for baseline Adult index changed the estimate of the effect of the dengue interventions from null to detrimental, with a borderline p value (0.04), although this analysis was not pre-specified and omitted one school due to lack of baseline data. The number of pupae per person in this study appeared to be lower in the DEN and DIADEN arms (0.04 and 0.05, respectively) compared to the other arms (DIA = 0.18 and CON = 0.36), a reduction of about 94%, although not significant (p = 0.11). The pupae per person values observed in schools in the CON arm were similar to those found in households in a cluster-randomized trial in a nearby municipality [67].

One reason that no apparent benefit of interventions was detected on the adult mosquito population was that they could have flown in from nearby breeding sites that were not targeted in the study. Most schools had 1–5 households nearby, potentially providing mosquito breeding opportunities (Table 1). Few cryptic breeding sites were found in the schools. There were no storm drains in any of the schools; all rainwater gutters were inspected and were negative; septic tanks only contained *Culex* larvae; and all elevated tanks were treated. In a separate paper we have reported on mosquitoes collected in households near schools [37]. The indoor resting density of *Ae*. *aegypti* females was average 2.7–3.0 mosquitoes (maximum 22 mosquitoes) per 10 minutes collection effort. All of the above indicate that adult mosquitoes collected in schools probably did not originate from the school area. Insecticide susceptibility tests carried out before the trial confirmed that *Ae*. *aegypti* from the study area were susceptible to pyrethroid insecticides, including deltamethrin which was used in the curtains (S1 Table). Assuming that the curtains were indeed effective, a difference between arms should have been observed in this situation. Thus, the absence of resistance does not explain the observed results. Furthermore, it is interesting to note that the overall female density of *Cx*. *quinquefasciatus* was 3–12 times higher than *Ae*. *aegypti* ([36], S1 Fig), but there were no significant differences between arms (S1 Fig). It is likely that curtains did not provide an effective physical and chemical barrier preventing mosquito entry into school classrooms. In fact, it was not possible to cover all possible mosquito entry sites to classrooms with curtains. Another observed problem was that the polypropylene material of these curtains was very light and a slight breeze could make curtains move away from the open windows allowing mosquitoes to enter. This was partially amended by adding weights to the lower ends of curtains to keep them hanging straight. The physical integrity of the curtain material was also observed to deteriorate very quickly with exposure to wind and sun potentially allowing mosquitoes to enter. The lack of complete blockage by insecticide treated window and door curtains has been observed in other studies, notably in Thailand where open housing structures were suggested to reduce the likelihood of mosquitoes making contact with insecticide-treated curtains [72]. On the other hand, a significant reduction in BI was observed in urban households in Girardot, Colombia using insecticide-treated curtains in windows and doors, although these differences were not significant for the pupae per person index (proxy for adult vector densities) [67]. This was also explained by the incomplete coverage of all household points of potential mosquito ingress and egress, allowing some mosquitoes to avoid contact with treated materials. However, an additional successive intervention in the Girardot study consisting of water storage container covers made from the same insecticide-treated material as the curtains showed a significant reduction in pupae per person [67]. As in our study, this demonstrates the importance of combining multiple vector control interventions targeting different stages of the mosquito life cycle.

Although there were, in general, few cases of dengue during the study period, we found a significantly higher rate of absence due to probable dengue in La Mesa than in Anapoima (0.03 vs. 0.019 episodes/student/year; p = 0.03 and 0.15 vs. 0.008 days/student/year; p = 0.047). The reasons for these geographical differences are unclear, but other studies have shown high spatial and temporal variability in occurrence of dengue infection in schools [73, 74]. Population density is a possible explanation that favors dengue transmission, due to the higher human-vector contact. The overall human density in La Mesa was close to double that of Anapoima (La Mesa: 200 persons/km^2^; Anapoima: 102 persons/km^2^). In addition, the number of pupils in the schools in La Mesa was higher than that of the schools in Anapoima.

It is possible that children in the DIADEN arm had a higher knowledge of either disease compared to children in other arms due to the educational intervention. This could have contributed to the observed lower BIs, through more vigilant and knowledgeable pupils who were active in cleaning up garbage around schools. Similar results have been found in Ecuador [75], where pupal indices were lower in schools which received integrated intervention strategies (including dengue education, patio and container clean-up campaigns through community empowerment and social mobilization) compared to the conventional dengue prevention government program (including routine temephos larval control and reactive/targeted deltamethrin and malathion fogging).

From the above it is clear that combinations of interventions are needed to reduce not only larval indices, but also the number of pupae and adult mosquitoes, which eventually could have an effect on dengue transmission [76].

### Diarrhea

The interventions targeting diarrhea risk factors were effective at providing cleaner water to pupils. The mean *E*. *coli* concentrations were 78% lower in schools receiving diarrhea interventions (DIA and DIADEN arms) compared to schools in the CON and DEN arms (p<0.05). Furthermore, the proportion of *E*. *coli* contaminated water samples were also significantly lower in the DIA and DIADEN intervention arms. These results mean that provision of water filters, hand washing, cleaning and covering of water storage containers can effectively reduce exposure to fecally contaminated water in school children in these settings. These interventions alone or in combination with other interventions have shown to reduce water contamination and diarrheal disease in a variety of settings. Water filters implemented in households in Bolivia provided 100% coliform-free drinking water (based on 96 water samples) and significantly reduced diarrheal disease in children less than 5 years of age [77]. In Cambodia, water samples taken from intervention households where two different kinds of water filters (ceramic with or without iron enrichment) had been installed showed that 37% and 40%, of samples, respectively were free of *E*. *coli*, and, in contrast, 85% of water samples from control households were considered higher risk (≥101 CFU/100 mL *E*. *coli*) [78]. The Cambodia study showed that, although only about 40% of water samples were completely free from *E*. *coli*, households using filters had significantly less diarrheal disease than control households. In the current study in Colombia, as many as 60–66% of water containers were free of *E*. *coli* in the arms receiving DIA interventions (Fig 4). Pupils in schools in a water-scarce environment in Kenya receiving water, sanitation and hygiene (WASH) improvements showed a reduction in diarrhea incidence and days of illness compared to control schools [79]. Despite this reduction in disease incidence there were no significant effects on overall pupil absence [80], indicating that other reasons for absence were common in that setting.

The interventions targeting diarrhea risk factors in our study did not appear to have an effect on school absence. There were no significant differences between arms in school absence due to any reason, due to any disease, or due to diarrhea. The reason for no apparent effect on absence due to diarrhea could be that these constituted a relatively minor proportion of the absences. Of the 25% of all absences that were due to illnesses, only 8% were due to diarrhea. The overall incidence rate of diarrhea was 0.3 absence days per pupil per year or 0.1 absence episodes per pupil per year. These incidence rates could have been too low to detect significant differences. In other potentially comparable studies, there were 5.9 absence days due to diarrhea for children in schools in Bogotá, Colombia [81] and 0.6 absence episodes per child per year in state schools in Spain [82]. The first is almost 20 times higher and the second about six times higher than what we found. The incidence rate reported in the Spanish study also included respiratory diseases and influenza, so diarrhea alone would be lower and potentially closer to the figures in our study. Nonetheless, it is not clear why there is such a large difference and normally one would expect higher diarrhea incidence in rural areas than in urban areas [83]. A systematic literature review of diarrhea incidence estimates from 139 low and middle-income countries showed an average of 2.9 diarrhea episodes per child per year in 2010 [84]. This is 29 times higher than in our study, but this estimate is for children under 5 years old and would naturally be higher than in school-aged children.

### Limitations

Some limitations of this study are worth mentioning. The interventions were exclusively implemented in schools, with no attempt to control exposure in households and communities. Schools-based interventions only target the time at risk when students are in school. It is possible, therefore, that the lack of an effect on the primary diarrhea endpoint was a result of external factors outside the school environment. In retrospect, it was optimistic to base the sample size calculation on a 75% reduction in diarrhea incidence by the interventions targeting water, sanitation, and hygiene factors, especially since there were no interventions outside the school.

Another issue is the school hours and children’s time at risk. In this location, children were in school from 07:00–13:00. Mosquito collections done in a nearby community in 1978–1979 found that *Ae*. *aegypti* had two biting peaks at 10:00–11:00 and at 16:00–17:00 [42]. In the current study, biting times were not studied. However, if the mosquito has not changed its behavior it is possible that children were also exposed when they were not at school, highlighting the importance of simultaneous community interventions.

Disease assessment based on reporting by parents or non-clinical observations by project staff (as done regularly in this study) may not be sufficient to assess an impact on a disease endpoint, particularly in the case of dengue. Another confounding factor is that some children might have gone to school despite being ill. This was not measured in this study, but should be accounted for in future work, for example by observations of fever or other obvious symptoms by teachers and other school staff. Information about general school absence is lacking in Colombia and no published information was found on children going to school sick. Future studies should include better epidemiological endpoints, with laboratory confirmation where necessary, although these may be expensive. Due to the large spatial-temporal variations in dengue, large randomized controlled trials are needed to find suitable interventions and combinations of interventions for each setting. As the new Dengvaxia vaccine, which has been rolled out in several countries, is not 100% effective [85], integrated interventions, including vector control, will remain important for future dengue control.

There are potential practical and statistical implications of duplicating container lids or nets in the DIA and DEN interventions. Lids and nets on water containers target both diarrhea and dengue outcomes, especially water quality and larval indices. The implications of this is that the observed better water quality in DIA schools compared to DEN schools (Fig 4) would more likely be due to water filters and container cleaning in the DIA schools rather than the joint lids and nets interventions. Similarly, the lower BI’s in the DEN and DIADEN schools compared to DIA schools (Fig 3) would more likely be due to the additional pyriproxyfen and garbage clean-up campaigns in the DEN schools rather than the lids and nets.

### General

This study was conducted in rural areas because they are often neglected in terms of national health policies. Although national dengue control primarily takes place in urban areas, dengue transmission also occurs in rural areas [86, 87]. In Colombia between 13–29% of dengue cases are reported from rural areas [30–32]. We also found a high prevalence of DENV infected *Ae*. *aegypti* in the study area; 62% of mosquito pools were positive, the estimated individual mosquito infection rate was 4%, and in 74% of the examined households DENV-positive mosquitoes were present [37].

Relatively few studies have investigated the effect of health interventions on dengue or diarrhea in schools [e.g. 80, 88]. School children participating in school-based interventions may bring health messages back home to their parents and diffuse them through the wider community [48]. We will report on the effect of the educational components on knowledge, attitudes and practices in students and their parents, as well as teachers, in a subsequent publication.

This study was not designed to evaluate the effect of specific single interventions on outcome measures. The overall effect of each of the two sets of disease-specific interventions was of interest here. Reliance on a single intervention to control vector borne diseases has often been ineffective and combinations and integration of interventions are recommended by the WHO [76]. Future research using similar integrated interventions in schools should also involve parents and the surrounding communities. The effect of school-based integrated interventions should also be implemented in urban areas to assess the effectiveness and sustainability of interventions in settings with higher human and vector densities and more complex infrastructure and human dynamics.

Finally, the results of this study will hopefully encourage development of policy recommendations for this school-based approach. The appropriate combination of interventions should be location-specific, effective, acceptable, and affordable. Therefore, the selected combination of interventions must be tested first before scale-up. Research on joint school and community-based interventions should be carried out in different settings to allow clear location-specific policy recommendations.

## Conclusions

A cluster randomized control study was carried out in rural primary schools in Colombia with the aim to reduce diarrheal disease and dengue entomological risk factors. Integrated control of these two diseases is justified because of common risk factors existing in potentially contaminated drinking water stored in containers which may also harbor immature dengue vectors. Integration of interventions could, therefore, effectively control disease outcomes in cost-efficient ways. Two sets of interventions, one targetting diarrheal risk factors and the other dengue risk factors, were implemented. The results show that schools with dengue interventions had a significantly lower Breteau index (larval breeding) and schools with diarrhea interventions had significantly cleaner drinking water compared to schools without these interventions. There were no significant differences in pupil school absence due to diarrhea (absence used as proxy for incidence) or density of adult mosquitoes. The reason for no apparent effect on absence due to diarrhea could be that pupils were exposed to risk factors at homes and elsewhere, which these school-based interventions did not target. No effect on adult mosquito populations were likely due to a failure of insecticide-treated curtains and mosquitoes flying in from nearby untreated breeding sites. The study highlights the importance of combining several vector control interventions targeting different stages of the mosquito life cycle. Overall, the study suggests that integrated approaches to disease control in school settings can be effective in reducing disease risk factors in the school environment, but that simultaneous interventions in communities must be emphasized. The appropriate combination of interventions must be location-specific, effective, acceptable, and affordable and tested before scaling up and providing policy recommendations.

## Supporting Information

S1 Checklist CONSORT Checklist. (DOCX)

S1 Fig Effect of interventions on density of female *Culex quinquefasciatus*. Mean adult mosquito numbers (horizontal bars) in rural primary schools in four arms receiving either 1. Dengue, 2. Diarrhea, 3. Denguediarrhea (both) interventions, or 4. Control (no intervention). Each plot symbol is one school (A = Anapoima, M = La Mesa). There was no significant effect of the interventions (ratio 0.79, 95% confidence interval 0.33–1.83, p = 0.564). The number of *Cx*. *quinquefasciatus* / *Ae*. *aegypti* collected per school with the same sampling effort were as follows: DEN: 517/45; DIA: 293/47; DIADEN: 192/66; and CON: 479/60.
(TIF)

S1 Table Susceptibility of female *Aedes aegypti* in the study area. Three replications of 80 females each were carried out for each insecticide and location. Diagnostic doses and times were as follows: Permethrin: 6.25 μg/ml; 15 minutes. Deltamethrin: 6.25 μg/ml; 30 minutes. Lambdacyhalothrin: 6.25 μg/ml; 15 minutes. The guidelines of the Instituto Nacional de Salud of Colombia and CDC (2010) were followed.
(DOCX)

S1 Dataset Data on diarrhea absences. (CSV)

S2 Dataset Description of variables in S1 Dataset. (CSV)

S3 Dataset Data on dengue absences. (CSV)

S4 Dataset Description of variables in S3 Dataset. (CSV)

S5 Dataset Data from immature mosquito collections. (CSV)

S6 Dataset Description of variables in S5 Dataset. (CSV)

S7 Dataset Data on water quality. (CSV)

S8 Dataset Description of variables in S7 Dataset. (CSV)

S9 Dataset Dataset of adult mosquito collections and variable description. (XLSX)
