# Supplementary figures and images for: A Cluster-Randomized Controlled Trial to Reduce Diarrheal Disease and Dengue Entomological Risk Factors in Rural Primary Schools in Colombia
Source: PLoS Negl Trop Dis. 2016 Nov 7;10(11):e0005106. doi: 10.1371/journal.pntd.0005106 (PMC5098800; doi:10.1371/journal.pntd.0005106)

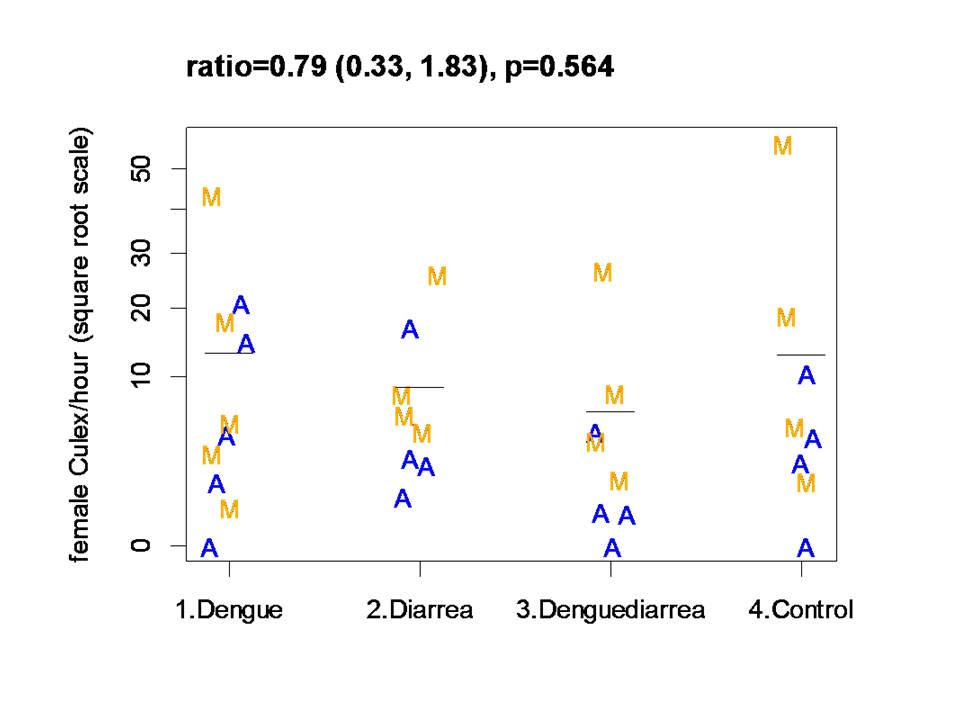

Supplement: S1 Fig — Mean adult mosquito numbers (horizontal bars) in rural primary schools in four arms receiving either 1. Dengue, 2. Diarrhea, 3. Denguediarrhea (both) interventions, or 4. Control (no intervention). Each plot symbol is one school (A = Anapoima, M = La Mesa). There was no significant effect of the interventions (ratio 0.79, 95% confidence interval 0.33–1.83, p = 0.564). The number of Cx. quinquefasciatus / Ae. aegypti collected per school with the same sampling effort were as follows: DEN: 517/45; DIA: 293/47; DIADEN: 192/66; and CON: 479/60. (TIF) [file pntd.0005106.s002.tif]
