# Supplementary material for: A Cluster-Randomized Controlled Trial to Reduce Diarrheal Disease and Dengue Entomological Risk Factors in Rural Primary Schools in Colombia
Source: PLoS Negl Trop Dis. 2016 Nov 7;10(11):e0005106. doi: 10.1371/journal.pntd.0005106 (PMC5098800; doi:10.1371/journal.pntd.0005106)
Supplement: S1 Table — Three replications of 80 females each were carried out for each insecticide and location. Diagnostic doses and times were as follows: Permethrin: 6.25 μg/ml; 15 minutes. Deltamethrin: 6.25 μg/ml; 30 minutes. Lambdacyhalothrin: 6.25 μg/ml; 15 minutes. The guidelines of the Instituto Nacional de Salud of Colombia and CDC (2010) were followed. (DOCX) [file pntd.0005106.s003.docx]

**S1 Table. Susceptibility of female *Aedes aegypti* in the study area.** Three replications of 80 females each were carried out for each insecticide and location. Diagnostic doses and times were as follows: Permethrin: 6.25 μg/ml; 15 minutes. Deltamethrin: 6.25 μg/ml; 30 minutes. Lambdacyhalothrin: 6.25 μg/ml; 15 minutes. The guidelines of the Instituto Nacional de Salud of Colombia and CDC (2010) were followed.

| **Population/Location** | **Insecticide** | **Numbers tested** | **Numbers dead after diagnostic time** | **% mortality** |
| --- | --- | --- | --- | --- |
| La Mesa - rural | Permethrin | 240 | 240 | 100 |
|  | Deltamethrin | 240 | 240 | 100 |
|  | Lambdacyhalothrin | 240 | 240 | 100 |
| Anapoima - rural | Permethrin | 240 | 240 | 100 |
|  | Deltamethrin | 240 | 240 | 100 |
|  | Lambdacyhalothrin | 240 | 240 | 100 |
| Anapoima - rural village (semi-urban) | Permethrin | 240 | 240 | 100 |
|  | Deltamethrin | 240 | 240 | 100 |
|  | Lambdacyhalothrin | 240 | 240 | 100 |
| La Mesa- rural village (semi-urban) | Permethrin | 240 | 240 | 100 |
|  | Deltamethrin | 240 | 240 | 100 |
|  | Lambdacyhalothrin | 240 | 240 | 100 |

CDC. 2010. Guideline for Evaluating Insecticide Resistance in Vectors Using the CDC Bottle Bioassay. Centers for Disease Control and Prevention, Atlanta, USA. <https://www.cdc.gov/parasites/education_training/lab/bottlebioassay.html>.
